# Thiersch graft follow-up with narrow band imaging for acquired atresia of the external auditory canal: Canaloplasty with Thiersch graft versus vascularization evaluated with narrow band imaging

**DOI:** 10.17305/bjbms.2021.6876

**Published:** 2022-03-02

**Authors:** Annalisa Pace, Valeria Rossetti, Irene Claudia Visconti, Alessandro Milani, Giannicola Iannella, Antonino Maniaci, Salvatore Cocuzza, Giuseppe Magliulo

**Affiliations:** 1 Department of Organi di Senso, Sapienza University of Rome; Rome, Italy; 2 Department of Scienze Chirurgiche, Sapienza University of Rome, Rome, Italy; 3 Department of Otorinolaringoiatria, University of Catania, Catania, Italy

**Keywords:** Acquired atresia, narrow-band imaging endoscope, cold white light endoscope, thiersch graft

## Abstract

Acquired atresia of the external ear canal (EAC) is defined as a narrowing caused by abnormal production of soft or bony tissue. EAC has two different phases, a wet one and a dry one. A computed tomography (CT) scan may show images where the soft tissue fills the EAC. Treatment with CT may be medical and/or surgical. The surgical technique most widely adopted is canaloplasty with a skin free flap. To the best of our knowledge, no previous article has reported data analyzing the vascularization of acquired atresia of the EAC and vascularization of the skin flap during follow-up with narrow-band imaging (NBI). This paper aimed to study post-surgical vascularization of the skin graft, with NBI endoscopy, to identify any eventual areas with less perfusion that may lead to degeneration and fibrosis. Patients suffering from acquired atresia of the external auditory canal, surgically treated in the Department of Organi di Senso of Sapienza University, from 2017 to 2020 were enrolled. All patients underwent anamnestic collection, physical examination, and mastoid CT. Pre- and post-operative otoendoscopic evaluations (at 1, 3, 6 and 12 months) were performed with both cold white light (CWL) and NBI endoscopic vision. 17 patients were enrolled in the study. Pre-operative otoendoscopic examination of CWL showed stenosis with a diameter <75% and a tympanic membrane not viewable in all patients. At 12 months of follow-up, 94% of patients had no recurrence of EAC stenosis. 88% of patients presented an adequate vascularization by NBI. Our study aimed to evaluate whether the NBI endoscopic view and the analysis of vascularization may be useful for improving the prognosis of patients surgically treated with canaloplasty and Thiersch graft for acquired atresia of EAC, concerning the single analysis using CWL endoscope.

## INTRODUCTION

Acquired atresia of the external ear canal (EAC) is a rare disease, mainly consequent to chronic otitis externa or media [1]. It is classified into two different phases: the wet phase (otorrhea and fullness) and the dry phase (progressive hearing loss).

The wet phase could be considered as the reversible phase where episodic inflammation and granulation may incur in fibrosis but could be interrupted with medical treatment. On the contrary, the dry phase is an irreversible condition for the EAC which presents a stenotic and not-discharging appearance [2].

Surgical management is the gold standard for the dry types and one of the techniques proposed is canaloplasty with Thiersch graft [1].

Paparella et al., in 1966, were the first to describe the surgical technique for the removal of dry types of acquired atresia of EAC and its repair using a Thiersch graft. Primarily, an excision of the fibrous tissue was performed, preserving the fibrous layer of the tympanic membrane (TM). The external auditory canal was enlarged with a burr and flattened to allow correct positioning of the skin graft covering the entire bony surface. Finally, packing is positioned to maintain patency [3].

Many authors subsequently modified this technique. For example, Katzke and Pohl covered the bony and deep area with the dissected stenotic tissue or covered the area of the TM with a free split skin graft [2,4].

McDonald et al. removed all abnormal skin tissue re-covering the bony structure with a skin graft and anchoring it to the conchal bowl [5].

In our experience, the best surgical method is to remove all the stenotic tissue, replacing it with a Thiersch graft that covers the entire area of exposed bone.

However, to the best of our knowledge, no papers have previously tested the status of graft vascularization with modern techniques.

At present, narrow-band imaging (NBI) is reported as a useful instrument, able to filter specific narrow-band light (415 and 540 nm wave-lengths) by penetrating tissues at different depths. Therefore, its filters can visualize tissue vascularization by selecting blue and green wavelengths, typical of hemoglobin, thus highlighting the microvascular texture [6,7].

NBI is mainly used to perform early diagnosis in the head and neck, since it can easily identify the hypervascular areas of pre-cancerous lesions. However, many studies reported its possible application for benign head and neck lesions too [8].

In the literature, two papers used this method for otological diseases, but neither reported data regarding the vascularization analysis of acquired atresia of the EAC or the revascularization of the Thiersch graft during follow-up [9,10].

The aim of the present study was to evaluate the vascularization of the skin graft, tested with NBI endoscopy to check areas with lower perfusion that may lead to degeneration and fibrosis.

## MATERIALS AND METHODS

This study evaluated patients affected by acquired complete stenosis of EAC, surgically treated in our Department from 2017 to 2020. All subjects enrolled underwent canaloplasty and Thiersch graft performed by one of the authors (G.M.). Anamnesis, clinical symptoms, pre-operative otoendoscopic signs, and the results of a high-resolution mastoid computed tomography (CT) scan were collected. Pre-surgical Pure Tone Audiometry (PTA) showed conductive hearing loss classified as: normal (0-20 dB HL), mild (20-40 dB HL), moderate (40-70 dB HL), severe (70-90 dB HL), and profound (above 90 dB HL) [11].

The pre-operative otoendoscopic evaluation was performed with both cold white light (CWL) and NBI endoscopic viewing. The flexible video endoscope adopted was 3.4 mm HD, coupled with the NBI system (ENF-VH^©^ Olympus, Tokyo, Japan).

NBI is a technology that tests the macroscopic characteristics of a mucosal lesion and its vascularization. It filters two exclusively selected wavelengths (415 and 540 nm) corresponding to blue light and green light. The latter has a specific absorption peak for hemoglobin, ensuring the emphasis of blood vessels that appear dark blue if located at the epithelial level or green if located at the submucosal level.

One author (VR) performed and graded the diameter of the stenosis with CWL classifying it as Grade 1: >25% of diameter reduced; Grade 2: 25-75% of diameter reduced; Grade 3: <75 of diameter reduced. The same author evaluated the grade of stenosis vascularization with NBI. It was adopted using a semi-quantitative grading system (0-2) proposed by Devesa et al. [3] who classified vascularization into 3 grades (0-2). 1 =absence of vascularization; 2= poor vascularization; 3= normal or abundant vascularization. Images were blinded and re-evaluated by two different authors (A.P; G.M). Another author (A.M) extracted the average of the scores.

All patients were surgically treated with canaloplasty and reconstruction using Thiersch graft. The approach was endoaural. The skin incision was made 1-2 mm lateral to the fibrous plug. The fibrous tissue was removed by blunt dissection from the anterior and posterior bony canal. Posteriorly, the fibrous plug was elevated reaching the fibrous annulus. This maneuver enables us to identify an avascular plane between the meatal fibrosis and the fibrous layer of the TM. The dissection proceeded until the entire lesion had been removed. If the patient had a perforation or an ossicular chain disruption, myringoplasty, and ossiculoplasty were performed at the same time. The bony wall canal was surgically made cylindrical, adopting diamond burrs. Thiersch grafts were used to cover the external auditory canal from the retro-auricular region. The surgeon performed packing in the ear canal with Gelfoam and with a thin sheet of Silastic.

Follow-up was done 1, 3, 6, and 12 months after surgery with otoendoscopic CWL and NBI.

Since, to the best of our knowledge, there is no standard criterion to define the recurrence of stenosis, this was reported when the patient showed a narrowing of EAC ≥25%.

### Ethical statement

This study was approved by the “Sapienza” Ethical Committee (RIF.CE.6268,) following the principles of the Declaration of Helsinki. Informed consent was signed by each patient enrolled in the study.

## RESULTS

About 20 patients were enrolled in the study (12 females and 8 males), with an average age of 35.6 years. Three patients were excluded from the study: two were lost to follow-up and one patient was treated with canaloplasty without Thiersch graft. Patients’ characteristics and PTA results were reported in Table 1.

**TABLE 1:**
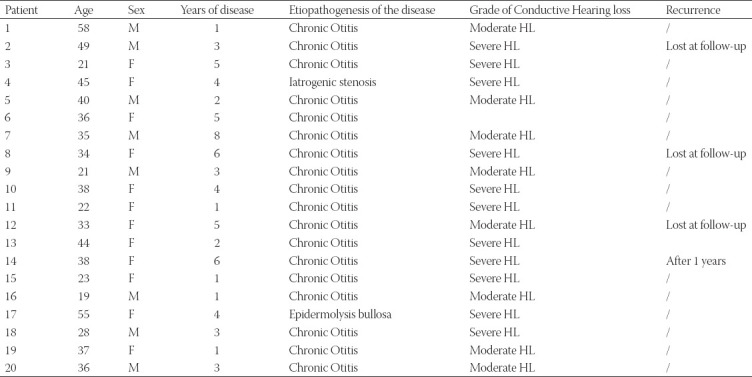
Patient characteristics, clinical conditions, and pharmacological treatment

Acquired atresia of the external auditory canal was due to chronic otitis in 76% (13/17): 5 had previous middle otitis, while the others had an external one. One stenosis was induced by bullous epidermolysis of EAC (6%), and 18% (3/17) had an iatrogenic cause (Table 1).

**FIGURE 1:**
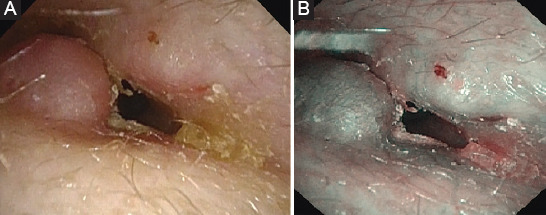
Right ear, pre-operative view. (A). Cold White Light. Stenosis <75% of the lumen of the external auditory canal (B). Narrow band imaging. Stenosis <75% of the lumen of the external auditory canal with Grade 3 vascularization.

CWL pre-operative otoendoscopic exam showed stenosis with a diameter <75% (Table 2) and a picture of an obscuring TM in all patients (Figure 1). NBI exam revealed patterns of vascularization as summarized in Table 3.

**TABLE 2:**
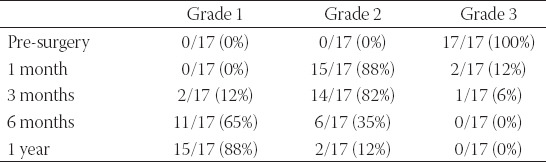
Grading of stenosis tested with cold white light at 1, 3, 6 months, and 1 year

**TABLE 3:**
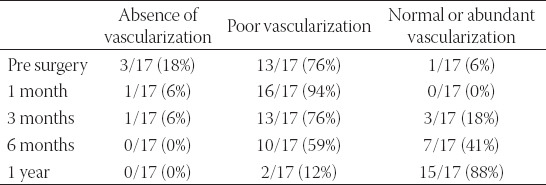
Grading of stenosis tested with cold white light at 1, 3, 6 months, and 1 year

Intraoperative findings showed a predominant narrowing of the bony canal in 71% (12/17) of patients while the others had mainly a soft abnormal growth.

**FIGURE 2:**
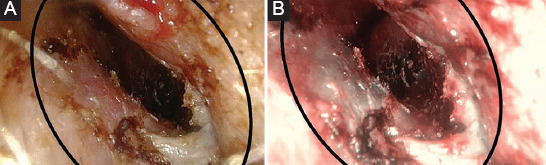
Right ear, post-operative view. (A). Cold White Light. Follow-up at 3 months. Thiersch graft in the external auditory canal (circle). (B). Narrow band imaging. Follow-up at 3 months. Thiersch graft in the external auditory canal (circle) with Grade 2 vascularization.

**FIGURE 3:**
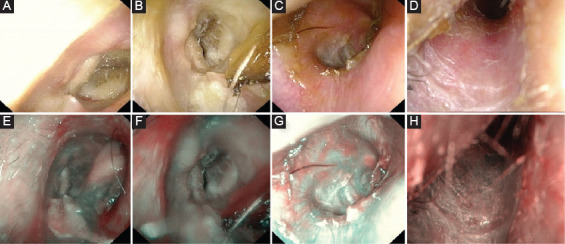
Post-operative Follow-up. Right ear. (A) Cold White Light at 1 month: stenosis >25%; (B) Cold White Light at 3 months: stenosis >25%; (C) Cold White Light at 6 months: complete healing of the Thiersch graft; (D) Cold White Light at 1 year: complete healing of the Thiersch graft; (E) Narrow Band Imaging at 1 month: vascularization Grade 1; (F) Narrow Band Imaging at 3 months: vascularization Grade 2; (G) Narrow Band Imaging at 6 months: vascularization Grade 3; (H) Narrow Band Imaging at 1 year: vascularization Grade 3.

After surgery, an otoendoscopic exam with CWL and NBI showed a modification of the diameter of the stenosis and the vascularization (Figures 2 and 3).

Stenosis of the EAC evaluated with CWL before surgery showed that 88% of patients had a stenosis categorized as Grade 2 and 12% categorized as Grade 3. No subject had a Grade 1 stenosis. One year later, there was an inversion of clinical evidence with 88% of patients in Grade 1 and 12% in Grade 2 (Table 2)

Regarding vascularization evaluated with NBI, before surgery vascularization was absent in 18% of patients, poor in 76%, and normal in 6%. It improved during follow-up at 3 and 6 months, reaching 88% of patients with normal vascularization after 1 year and only 12% with a poor one. No subjects reported absent vascularization (Table 3).

During follow-up, 3 patients showed a small portion with less vascularization. They were treated with silver nitrate and followed weekly for 1 month. All of them presented a normal final vascularization at 1 year.

At the 12 month follow-up, 94% (16/17) of patients had no recurrence of EAC stenosis. The patient who reported recurrence showed a progressive closure of the external auditory canal with a closure ≥75% after 1 year. She had a history of chronic middle ear otitis with multiple episodes starting 3 months after surgery.

## DISCUSSION

Acquired external auditory canal stenosis is an uncommon disease with different causes such as infection, trauma, tumor, and inflammation [4,12]. Its common presenting features are a blind skin-lined canal lateral to the TM while pure tone audiometry reveals a conductive hearing loss [13].

In our series, in accordance with the literature, the most common cause was chronic otitis (76%). The development of EAC stenosis may be connected to a subepithelial infiltration of inflammatory cells and edema related to the chronic inflammatory status, which induces a fibrotic change of the tissue [14]. To the best of our knowledge, there is not a defined classification of the grade of stenosis (related to the diameter). Therefore, we preferred to adopt criteria that allowed us to better define the pre-operative occlusion and the post-operative status of EAC during follow-up. The classification was based on the diameter of stenosis and surgical management was considered mandatory in cases of chronic stenosis with an occlusion <75% and in cases of moderate-severe conductive hearing loss.

PTA showed that the patients enrolled had moderate to severe conductive hearing loss. This is in line with the literature where a 30±40 dB air±bone gap is reported in the pre-operative analysis [2].

In our experience, the best surgical technique proposed is canaloplasty with Thiersch graft reconstruction. The latter is a dermo-epidermic partial-thickness, semitransparent skin graft positioned in the EAC to re-epithelialize the area of skin removed from the stenosis with a highly effective procedure and a shorter post-operative recovery time [15]. The good outcomes obtained with this type of management are confirmed by our results that showed a low incidence of recurrence (6%), in line with that reported by Drossaert et al. (9%) [16].

Since the fibrotic status of EAC stenosis was always assumed, it was never proven. Therefore, we analyzed preoperative cases, performing CWL to assess the grade of stenosis and NBI to check the vascularization. A CWL otoendoscopic view can estimate the grading of the stenosis but gives no information about skin vascularization. At present, the NBI system is used to diagnose malignant and inflammatory diseases in gastroenterology, urology, the tracheobronchial tree, and in head and neck patients. Its optical filter selects the specific passage of light of certain wavelengths, reducing light scattering and enhancing blood vessel visualization [17]. Moreover, Devesa et al. reported that NBI otoendoscopy was superior to CWL for visualizing vascularization findings [9]. These authors performed it in patients with perforation of the TM, while previously Zhang et al. had employed it in cholesteatoma patients [18].

In our study, NBI confirmed the fibrotic status of EAC revealing a prevalence of poor or absent patterns of vascularization in the stenotic ears. Moreover, it demonstrated how vascularization improved during follow-up, reaching a normal status in 88% of patients at 1 year. It appears probable that the remaining skin of the EAC, in contact with the Thiersch graft, allows correct re-vascularization without fibrosis or sub-epithelial edema and a low risk of recurrence. Furthermore, the single case of recurrence reported was complicated by middle ear disease that induced an inflammatory status of the Thiersch graft as well.

In the literature, it is estimated that in 60% of patients ears return to normal while recurrence is reported in 20%. However, these data are variable since there is a large range of follow-up duration [19].

NBI represents a semi-objective method that provides a picture of graft re-vascularization and checks the areas with lower perfusion that may incur degeneration and fibrosis. The decision to remove the avascular areas or refresh the most vascularized ones may be made.

This study was designed as an exploratory evaluation to investigate the possible use of NBI for improving post-operative outcomes in patients surgically treated for acquired atresia of the EAC. However, based on the results obtained, the authors intend to carry out a formally dimensioned study with adequate statistical power.

## CONCLUSION

Acquired atresia is a rare disease that can be surgically treated. One type of surgical management is canaloplasty with Thiersch graft reconstruction. However, no authors have tested its vascularization during follow-up. Our study evaluated how NBI may be a superior method, compared to CWL, for evaluating the status of the graft and may be relevant in the decision-making process.
